# Intelligent Analysis of Exercise Health Big Data Based on Deep Convolutional Neural Network

**DOI:** 10.1155/2022/5020150

**Published:** 2022-06-28

**Authors:** Cui Cui

**Affiliations:** Department of Sports, Huanghe Jiaotong University, Jiaozuo, Henan 454950, China

## Abstract

In this paper, the algorithm of the deep convolutional neural network is used to conduct in-depth research and analysis of sports health big data, and an intelligent analysis system is designed for the practical process. A convolutional neural network is one of the most popular methods of deep learning today. The convolutional neural network has the feature of local perception, which allows a complete image to be divided into several small parts, by learning the characteristic features of each local part and then merging the local information at the high level to get the full representation information. In this paper, we first apply a convolutional neural network for four classifications of brainwave data and analyze the accuracy and recall of the model. The model is then further optimized to improve its accuracy and is compared with other models to confirm its effectiveness. A demonstration platform of emotional fatigue detection with multimodal data feature fusion was established to realize data acquisition, emotional fatigue detection, and emotion feedback functions. The emotional fatigue detection platform was tested to verify that the proposed model can be used for time-series data feature learning. According to the platform requirement analysis and detailed functional design, the development of each functional module of the platform was completed and system testing was conducted. The big data platform constructed in this study can meet the basic needs of health monitoring for data analysis, which is conducive to the formation of a good situation of orderly and effective interaction among multiple subjects, thus improving the information service level of health monitoring and promoting comprehensive health development.

## 1. Introduction

The core of a deep neural network is to realize computer modeling of complex input data by simulating the deep learning process of the brain and the extraction process of abstract knowledge. A deep neural network converts input data into low-order features, mid-order features, and high-order features in the network by multilayer hidden layers layer by layer until the final feature classification through a multilayer combination approach, and its powerful feature expression capability makes the learned features portray the input data more closely to the essence, thus facilitating the differentiation of targets [1]. So, features occupy an important position in deep neural network methods. In traditional machine learning algorithms, each dimension of the extracted features usually has a specific physical meaning, and the feature extraction algorithms rely heavily on specific tasks and corresponding databases, and their practical effects and generalization capabilities are difficult to be widely used. However, deep neural network methods integrate the feature extraction process into the end-to-end network optimization training process through deep feature combination, and the network can automatically learn and distinguish the importance of features during training, thus achieving automatic extraction of data features, and this method can be widely used in a variety of task scenarios. The human posture estimation algorithm can obtain the location information of the key points of the human body in the original input data, which are usually located at the skeletal joints of the human body [2]. This can be regarded as bagging in ensemble learning, which can effectively reduce the variance of the model, thereby alleviating the overfitting problem of the model.

Intelligent surveillance refers to the combination of computer vision technology and traditional digital surveillance systems, using computers to automatically detect, locate, and track targets in surveillance video and to automatically analyze the status of the targets. With the international community's attention to the field of security in recent years, the streets of various cities are littered with many digital surveillance devices, and the number is growing explosively [3]. Many around-the-clock monitoring devices make great reduction in the security threat, but the traditional monitoring equipment is only used to collect video images and also requires many security personnel to monitor the video in real time, consuming a lot of human resources and leading to visual fatigue caused by false alarm leakage. With the development of artificial intelligence technology, which makes it possible for machines to understand the image, the intelligent monitoring systems automatically analyze the video and detect abnormal behavior of people, greatly reducing the rate of false alarms and saving a lot of labor costs. This has now become a hot spot for research in the field of security.

Due to the inherent discrete nature of the digital video, object motion may not exactly align with the sampling grid of the digital image, so simple motion compensation cannot accurately predict the current encoding block. To address this problem, researchers have further proposed the concepts of the fractional precision motion vector and subpixel motion compensation. At MCP, the interpolation filter is used to interpolate the fractional pixel reference image, and the final prediction block can come not only from the whole pixel reference image but also from the fractional pixel image [4]. Research shows that the fractional pixel motion compensation technique can effectively use antialias and remove compression noise, thus effectively improving the coding efficiency of MCP. The core of fractional pixel motion compensation is to design a suitable interpolation filter so that the interpolated fractional pixel image can more accurately predict the current image. Most of the interpolation filters used in current fractional pixel motion compensation techniques are manually designed based on the assumption of the finite frequency band of the video signal, which has the characteristics of low computational complexity and simple implementation. However, these hand-designed filters have several problems as follows. The efficiency of data query and statistical work should be fully considered. Therefore, the index should be created according to the characteristics of student data query. First, because of the complex and nonsmooth content of the video signal, the traditional fixed interpolation filters cannot handle the diversity of the video signal. Secondly, traditional interpolation filters cannot handle the different quality of video content. Therefore, designing more adaptive and expressive interpolation filters is the core of fractional pixel motion compensation technology research, which is important to further improve the coding efficiency of MCP.

## 2. Related Works

Shin and Gierl analyzed the theory of student physical health promotion through controlled experiments, which laid the theoretical foundation for subsequent research in this area [5]. The study of student physical health big data started earlier in many developed countries such as the United States. Although the research is in the initial stage of development, with the introduction of a series of national guidelines and policies, the research results are becoming increasingly fruitful [6]. DeepPose defines the human posture estimation as a nodal point regression problem, using convolutional neural networks to directly regress the coordinates of each node, and the authors use the idea of the multistage cascade. Each stage is a 7-layer AlexNet network, the authors use the idea of the multistage cascade, the initial stage gets the coarse position of the nodes, and the rest of the stages optimize the node positions continuously by correction, i.e., from coarse to fine process [7]. The input of each optimization stage is the domain subgraph of the node positions outputted from the previous stage. The DEPOSE method achieved excellent results on various human pose public datasets and achieved the best results at that time, laying the foundation of deep learning in the field of human pose estimation. Subsequently, more deep learning-based pose estimation algorithms were proposed, typically represented by the convolutional pose machine proposed by Shin-En et al. and the stacked hourglass network proposed by Dong et al. Since convolutional neural networks can automatically learn features of objects and combine them, avoiding the difficulty of designing features manually, with the development of heterogeneous computing chips such as GPUs, training large convolutional neural networks has become possible [8]. There is no doubt that deep learning will be the dominant approach in the field of human pose estimation for some time to come.

Usually, the CNN network structure combines convolutional and pooling layers for computation. The convolutional layer extracts the features, and the pooling layer filters out the outstanding features from the amplified feature attributes to perform the dimensionality reduction operation and finally passes them to the fully-connected layer for classification [9]. The local perceptual field is an important part of the convolutional operation, and the original image is convolved using the convolutional kernel to extract features; the reduction of data dimensionality is mainly achieved through the operation of the pooling layer. CNNs have a significant role in image processing and understanding [10]. When selecting and creating an index, the size of the chart, the amount of data, and the frequency of use should be fully combined to avoid creating an index. Too many indexes will seriously affect the performance of database query. The main application of CNN processing is some recognition and classification of two-dimensional images and does not apply to this paper, and the EEG signal has the characteristics of temporal and spatial attributes, so the CNN needs to be applied to the EEG signal recognition because the convolution of the features does not contain temporal and spatial attributes, so the convolution kernel in the convolution layer needs to be designed to extract only spatial features or temporal features of the matrix [11]. A 16-dimensional weight vector is used to define the geometric authorized constraint term, the smoothing and sharpening of edges, and the action of this constraint term on the interpolated image through a Markov random field. Zemmal et al. use minimized mean square error estimates to fuse the estimates of edges along with two orthogonal directions [12]. Rahman et al. propose an edge-directed cubic convolution interpolation mechanism to self-adapt the edge structure of the image [13]. The interpolation process is guided by estimating the most significant edges.

This paper analyzes the purpose and performance sources of fractional pixel motion compensation, thus revealing that the purpose of fractional pixel motion compensation in video coding is not only to obtain high-resolution reference frames by interpolation but also to improve the coding efficiency of motion compensation. Based on this finding, this paper redefines fractional pixel motion compensation by defining the fractional pixel interpolation process as a conditional mapping process, i.e., the process of mapping from an integer pixel reference to the current block to be encoded based on the fractional pixel motion vector. This paper further extends this definition to bidirectional prediction and proposes a general definition of bidirectional fractional pixel motion compensation. In this paper, we propose a convolutional neural network-based algorithm for fractional pixel reference generation and a method for generating training data based on video sequences. To integrate the proposed convolutional neural network-based fractional pixel reference generation model into the HEVC reference software, this paper proposes more efficient motion estimation and motion compensation coding algorithms. This paper further gives a theoretical explanation of reversibility from the basic principles of signal processing. Based on the reversibility of fractional pixel interpolation, a training algorithm for fractional pixel interpolation is proposed in this paper. Based on the reversibility, an unsupervised training framework is designed in this paper, which can automatically generate fractional pixel samples by training. The work in this paper presents the first unsupervised training framework for fractional pixel interpolation, which fundamentally solves the training solution problem of fractional pixel interpolation.

## 3. Deep Convolutional Neural Network Intelligent Analysis of Sports Health Big Data

### 3.1. Deep Convolutional Neural Network Design

**Figure 1:**
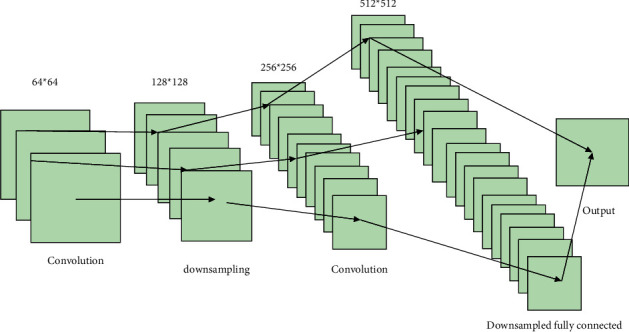
Convolutional neural network structure diagram.

Usually, the CNN network structure combines convolutional and pooling layers for computation. The convolutional layer extracts the features, and the pooling layer filters out the outstanding features from the amplified feature attributes to perform the dimensionality reduction operation, which is finally passed to the fully-connected layer for classification [14]. The model based on the time domain can achieve a maximum of 77.46%, which is about 5% lower than the accuracy index based on the frequency domain model. Further analyze the impact of attention methods on the way the input is divided. The local perceptual field is an important part of the convolutional operation, which uses a convolutional kernel to extract features from the original image; reducing the dimensionality of the data is mainly achieved through the pooling layer while retaining the most significant information. The feature of the local perceptual field allows CNN to extract features step by step through convolution kernels, and a simple pixel image is computed to obtain more information about it, while the feature of weight sharing allows CNN to better reduce the number of parameters, reducing the number of tuning parameters and the amount of computation. Each layer of the network has many independent neurons, and each neuron is connected to only some of the neighboring neurons. Feature information is obtained and enriched by the CNN structure of each layer, as shown in Figure 1.

The main role of the convolutional layer is to extract features, which is a fundamental part of the CNN, and the convolutional layer is the distinguishing mark of the CNN. The convolution kernel is a matrix of weights. By setting the size of the convolution kernel, the input original data are dotted with the weight matrix of the convolution kernel, then sliding to the next matrix range of the original data with the same size as the convolution kernel, and then continuing the dotting, so that multiple feature maps are obtained. The convolution operation not only magnifies some features of the original image but also has the function of noise reduction. In the image, the matrix value is calculated by the convolution kernel, because the difference between the value and the value is small, the average value of the surrounding pixels is taken, and the noise point is reduced. The value and the value have a large difference, which reflects the prominent places, and the features are enhanced. The lowest for *L*l is 44.35%. The corresponding overall accuracies of *L*l, *L*2, *L*3, *L*4, *L*5, *R*l, *R*2, *R*3, and *R*4, *R*5 are 44.35%, 50.36%, 61.33%, 61.38%, 60.45%, 62.47%, 60.14%, 60.71%, respectively, and 58.74%, 65.11%. The size of the convolution kernel is directly related to the computational effort, and if the size of the convolution kernel is set very large, the computational effort also doubles. Therefore, small size kernels are generally used. The stride and kernel size affects the size of the computed feature map. In the LeNET architecture, the 16 × 16 pixel map is subsequently convolved and computed through a 5-layer network architecture with a 3 × 3 kernel and a stride of 1, and the size of the output feature map is 14 × 14; with a stride of 2, the size of the output feature map is 7 × 7. The size of the feature map can be calculated by the following equation:

(1) $$\begin{array}{c} M=\frac{Y+Z}{S}-1,\\ f\left(x\right)=\frac{\text{exp}\left(-x\right)+\text{exp}\left(-x^{2}\right)}{\exp\left(x\right)-exp\left(-x\right)}. \end{array}$$

**Figure 2:**
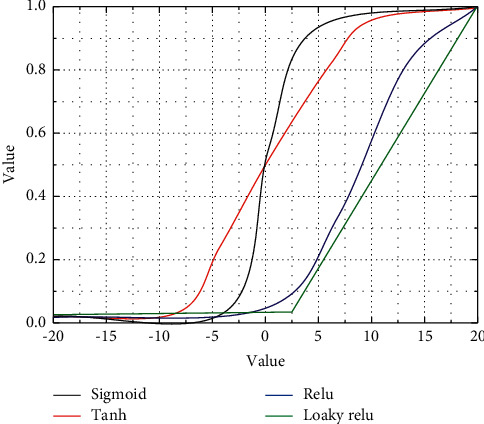
Activation function image.

The activation function is introduced in both traditional artificial neural networks and convolutional neural networks. The activation function not only simulates the characteristics of biological neurons but also has excellent mathematical significance, and the use of the activation function enhances the nonlinear mapping capability of the whole network, making the neural network capable of fitting almost any function, as shown in Figure 2.

The concepts of the loss function and objective function often appear in deep learning as conditions for network optimization. Many sources do not make this distinction and consider the loss function equal to the objective function. But strictly speaking, there is a difference between the loss function and the objective function [15]. On the other hand, the test set showed an upward trend after dropping to around 0.8, which is a typical overfitting phenomenon. The loss function is mainly used to measure the difference between the predicted value and the true value, i.e., the degree of model fitting, while the objective function is the function that the model needs to be optimized in the end, usually adding some regular term constraints after the loss function. For example, when the overfitting caused by the high complexity of the model needs to be considered, the objective function is usually defined as the form of

(2) $$\begin{array}{c} J=\text{max}\frac{1}{N}\sum_{i=1}^{N}L\left(y_{i},f\left(x\right)\right)-\lambda \left(f\right). \end{array}$$

The mathematical form of the absolute value loss function is the *L*1 parametrization and is therefore also called the *L*1 loss function. The *L*1 parametrization allows the weights to be sparse and facilitates feature extraction.

(3) $$\begin{array}{c} L\left(Y,f\left(x\right)\right)={\left(Y+f\left(x\right)\right)}^{2}. \end{array}$$

There are often many noisy signals among the acquired signals, and a certain kind of pure physiological electrical signal is often required for analyzing the physiological state. Effective separation or removal of these noisy signals is beneficial to the accurate analysis of deep learning models. Blind source signal separation can separate different signal sources, while wavelet packet transform can decompose the signal into different frequency bands. Unlike wavelet transform, wavelet packet transform has a more detailed portrayal of high-frequency signals.

**Figure 3:**
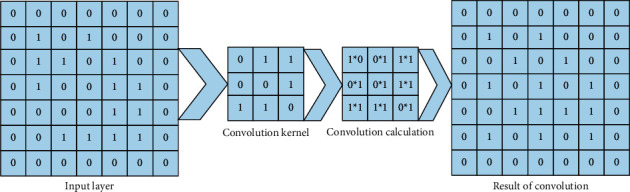
Convolution calculation diagram.

The shallow convolution kernel can extract the local features of the signal well, and as the network deepens, the deeper network can gradually abstract the shallow features into the overall features. Each convolution can perform a complimentary 0 operation on the matrix edges according to the size of the convolution kernel to prevent the feature map from becoming smaller in size after multiple layers of convolution.  Figure 3 shows the computation process of one two-dimensional convolution.

The black box is the pooled objects, mean pooling is to calculate the average value of all objects in the pooled area, and maximum pooling extracts the maximum value in the pooled area as features. In general, mean pooling can retain more preserved background information, and maximum pooling can retain more preserved texture information and eliminate the effect of feature position offset [16]. Compressing the feature map by pooling layer makes the feature map smaller and reduces the computational complexity on the one hand; on the other hand, it compresses the features to extract the main features and enhance the generalization ability of the model.

Using Dropout during training, the neural network will randomly discard a portion of the weights with a certain probability of not participating in the training of the model. Since some nodes will not participate in backpropagation, it is equivalent to generating multiple networks with different structures during training. At the end of model training, all parameters will be involved in forwarding propagation, and all parameters will be used to make common decisions, which can be regarded as bagging in integrated learning and can effectively reduce the variance of the model and thus alleviate the overfitting problem of the model.

Blind source signal separation is performed using the principle based on the maximum negative entropy, where the entropy value of a Gaussian signal is maximum for the equal variance of the signals. The entropy value is utilized in FastICA to measure the non-Gaussianity between signals, and the commonly used modified form of entropy is negentropy. Within the frequency domain-based feature fusion module, a combination strategy of both serial and additive operations is used. Since the network does not really learn the spatial distribution of human joints but only fits the training set with a huge number of parameters, the final model cannot be applied to the actual scene. The serial operation merges all local features to form a new global representation [17]. The core idea of the pairwise additive operation, on the other hand, is to give more information to the feature map without adding additional parameters to the network. Each feature extractor within the feature fusion module tends to bring an additional number of parameters, and too many network parameters can cause an overfitting phenomenon on a speech dataset with few samples. Also, since the two sets of global features correspond to the same dimensionality and the feature map space information is semantically similar, a pairwise summation operation is used within the feature fusion step instead of the conventional serial operation. Although the width of the network does not increase, the increase in the amount of information within each channel likewise helps to improve the classification capability of the network.

### 3.2. Intelligent Analysis System Design for Sports Health Big Data

To cooperate with the Education Bureau to conduct a comprehensive assessment and monitoring of primary and secondary school students' physical health and strengthen their physical fitness exercise, a set of perfect platforms based on Internet big data analysis technology is urgently needed to support recording and summarizing students' physical health data and to conduct a series of analysis processing and visualization presentation. In this way, the construction and planning of a big data platform for students' physical health are launched.

The construction of a big data platform for student physical fitness mainly focuses on collecting student physical fitness data and presenting the results statistically as two basic demands. First, the staff conducts accurate physical fitness tests on students through professional equipment. At the same time, the physical fitness test results of every student who takes the physical fitness test are accurately recorded or saved; secondly, among thousands of individual physical fitness test result records, the staff needs to statistically analyze the real and useful physical fitness test results and build a physical fitness big data platform for students by using modern computer technology [18]. The platform will input, add, modify, query, delete, compare horizontally and vertically, statistically analyze and process various test indicators, and compare various diagnoses of students in terms of individual scores and averages: individual posture diagnosis, overall posture diagnosis, and gap condition diagnosis. Queries include different physical fitness indicator values, scores and total scores of each assessment item indicator, physical fitness level of each assessment student, and each assessment indicator of mathematical statistics and graphical visualization.

**Figure 4:**
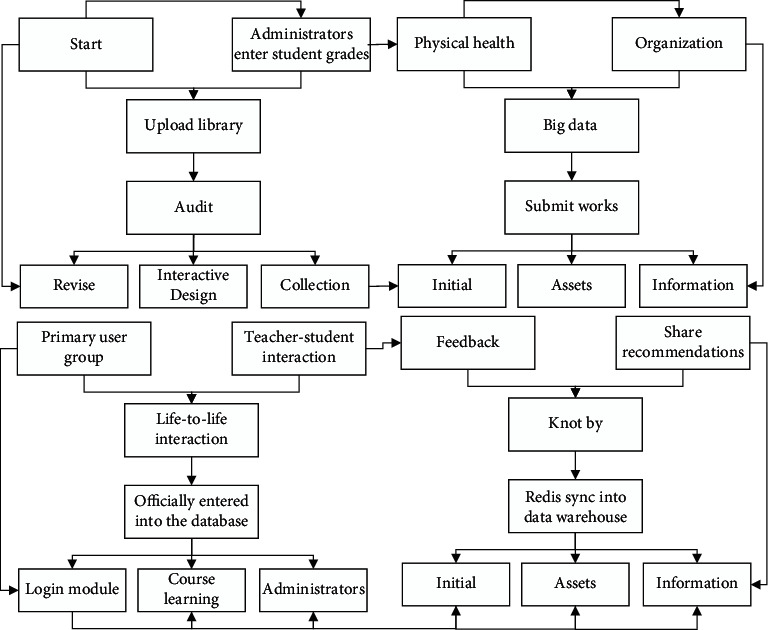
Flow chart of data management module.

The student physical health big data platform is dedicated to the government, schools, and other students and parents, and each platform user has certain roles and user groups according to their position in the social organization. According to the user structure of different functional roles, each user is put into a primary user group, secondary user group, and tertiary user group. Primary user groups are government managers. Secondary user groups are school administrators who are affiliated with government administrators. The tertiary user group refers to teachers who are affiliated with school administrators. The fourth-level user group refers to student users who are affiliated with classroom teachers and teachers, as shown in Figure 4.

First, after registration, the user's name and password are stored in the backend. Then, the user sends a username and password to the server during the system login process, and the server receives a request and verifies the username and password. Once the authentication is successful, the server generates a token by a specified algorithm, stores the token in Redis, and sends the token to the browser. The browser stores the token in local storage or session storage. The network can automatically learn and distinguish the importance of features during training, to realize automatic extraction of data features. This method can be widely used in various task scenarios. Finally, each subsequent request from the user will pass the token to the server via a cookie. When the server receives the request from the client, it parses the returned token to verify the user's information and compares the token value with the token value stored in Redis. If the verification is successful, the requested data are returned to the browser. If the token comparison is successful, the user is logged in; otherwise, the user needs to log in again because the login status is invalid. Each time the user logs back in, the token's expiration time is refreshed.

**Figure 5:**
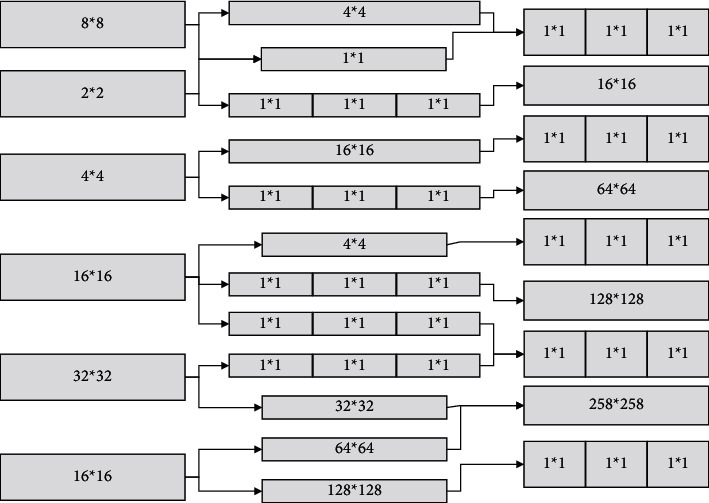
Data analysis entity E-R diagram.

This module integrates and analyzes the entered student physical health data to form a series of statistical charts and displays the charts in the foreground. It includes two parts, physical health evaluation and physical health diagnosis [19]. The evaluation and diagnosis are carried out for both individual and group objects, respectively. The submodule of evaluation is mainly composed of a single index score, comprehensive index score, single index grade, comprehensive index grade, the average score of the single index for male and female students, and an average score of the comprehensive index for male and female students. The functions of the physical health diagnosis submodule are score diagnosis, grade diagnosis, gap diagnosis, overall posture diagnosis, and individual posture diagnosis, as shown in Figure 5.

Data model warehousing analysis using physical data model is to change a logical data model directly into a data model that is compatible with the actual warehousing platform application. The following tasks need to be completed first in the process of designing the physical model for Hadoop-based data warehousing work. In this paper, we study the comprehensive testing statistics and multidimensional analysis of student physical health and other related data, so when we study and design the physical model in student data warehousing, we should fully consider the query and statistical efficiency of the data. So we choose to create indexes according to the characteristics of student data query, and when we select and create indexes, we fully combine the size of the chart and the amount of data and the frequency used to avoid. It also requires many security personnel to monitor the video in real time, which not only consumes a lot of human resources, but also easily causes false alarms and false alarms due to visual fatigue. The performance of database query is seriously affected by creating too many indexes.

**Figure 6:**
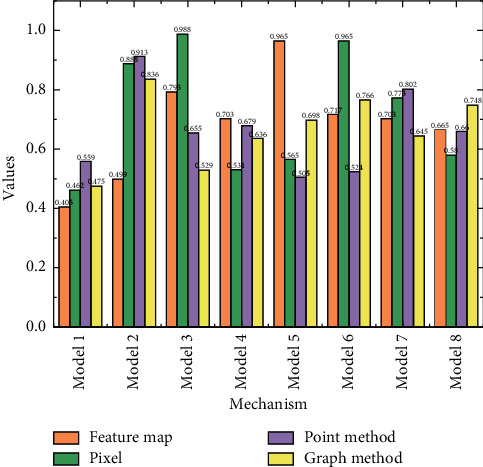
Analyzing the effect of attention mechanism on feature fusion.

The structured data mainly include basic student information data and student physical health monitoring data. These data are stored as database tables in MySQL database and then imported into the data warehouse using the Sqoop tool. The student basic information table (storing basic student information data), the student physical health monitoring data table (storing student physical health monitoring data), the class information table (storing class statistical analysis data), the school information table (storing school statistical analysis data), the information table (storing statistical analysis data), and the administrator information table (storing basic role information data) are listed respectively, as shown in Figure 6.

Again, the overall illustration shows that the feature fusion model based on frequency domain information performs better in terms of generalization ability, and when the attention mechanism is introduced, the time-domain-based model achieves a maximum of 77.46%, which is about 5% lower than the accuracy index of the frequency domain-based model [20]. Further analysis of the effect of the attention method on the input division method shows that the pixel point-based attention method is more capable of improving the generalization of the network when the division is based on time-domain information, while the feature map-based attention method outperforms the pixel point-based method when the model is divided based on the frequency domain. From the analysis of the number of attention modules, it can be obtained that, overall, the more the number, the more the network parameters, and the generalization ability of the model starts to decrease instead.

## 4. Analysis of Results

### 4.1. Algorithm Performance Analysis

We divide it according to the traditional method with a training set and a test set. In this case, we use 9 layers of data in the dataset as the size of the training set, and the remaining 1 layer is measured by the method, so the dataset has 10 rates. Our dataset is measured by the method of ten monitoring points, so the dataset has 10 classes corresponding to the data results of monitoring points *L*l, *L*2, *L*3, *L*4, *L*5, *R*1, *R*2, *R*3, *R*3, *R*4, *R*5; each class of data has 5000 samples, and we use 4500 data in each class as the training data for the training model and 500 data as the test set to verify the embodiment of the classification effect of the class.

**Figure 7:**
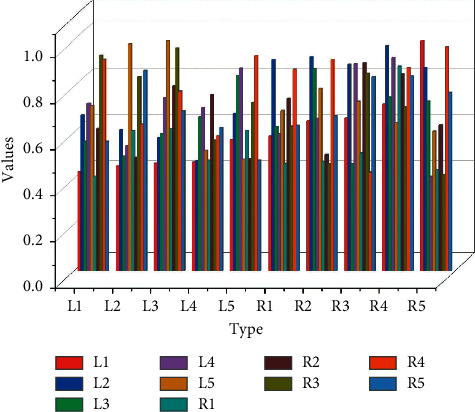
Overall average accuracy.

As shown in Figure 7 we can see the overall average accuracy of each monitoring point on the CNN model. It is the highest in *R*5, at the peak of 65.11%, while the lowest in *L*l is 44.35%. The corresponding overall accuracies for *L*l, *L*2, *L*3, *L*4, *L*5, *R*l, *R*2, *R*3, and *R*4, *R*5 are 44.35%, 50.36%, 61.33%, 61.38%, 60.45%, 62.47%, 60.14% 60.71%, and 61.23%, 60.87%, respectively. From the results, we can see that the overall average accuracy of each of the ten monitoring sites is above 44%. The interpolation filters used in the current fractional pixel motion compensation technology are mostly hand-designed based on the assumption of limited frequency band of the video signal and have the characteristics of low computational complexity and simple implementation. Figure 7 shows the group-level statistics and their standard deviations for each of the four MI tasks for each of the ten monitoring sites. Typically, *R*5 outperforms the others in the 2000 rounds of iterations, while *L*1 is the worst performer. The standard deviations are small, indicating that these accuracies are closer to the mean and stable.

**Figure 8:**
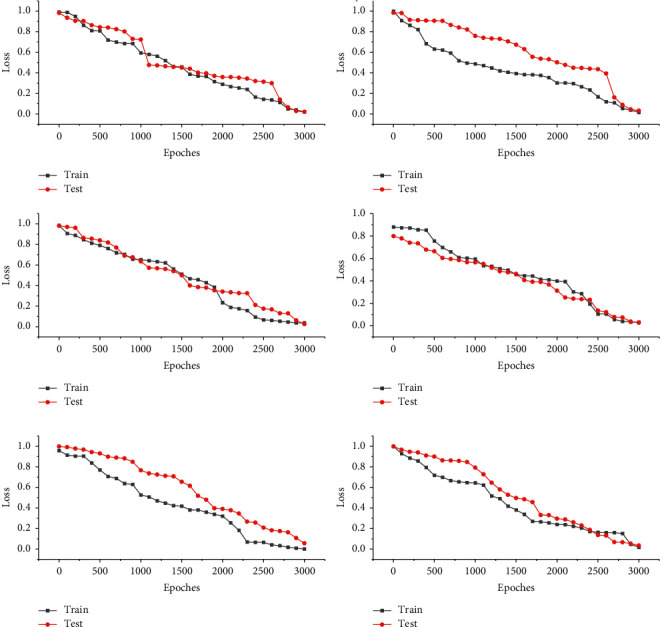
Error variation of training and test sets with the number of iterations.

The overfitting phenomenon of the DEPOSE model is more serious when there are not enough training samples, which makes it impossible to be applied in practical scenarios.  Figure 8 shows the decrease of loss error loss with the number of iterations when the DeepPose method is used to train the model, and the test set is validated every 500 iterations. After 50,000 iterations, the loss of the training set decreases to around 0.1, while the test set decreases to around 0.8 and then increases, which is a typical overfitting phenomenon. Since the network does not learn the spatial distribution of human joints, it only fits the training set with a huge number of parameters, so the final model cannot be applied to the actual scenario.

The biggest contribution of the DeepPose method is the idea of the multistage cascade, which gradually optimizes the result from coarse to fine. The initial stage starts with estimating the approximate position of the human joint points using CNN, and this stage effectively uses the overall information of the image. In the correction stage, the network uses the joint point coordinates obtained in the previous stage to select certain local areas in the original image based on these joint point coordinates, to obtain higher accuracy joint point coordinates. In this paper, we also use the idea of a multistage cascade to fuse the spatial constraint relationship between the nodes and combine the idea of increasing the network depth by the VGG network and the global average pooling layer proposed by the NIN132 network to design the global pose constraint network to solve the problems existing in DeepPose.

Usually, the output of the last convolutional layer of the network is a feature map that has been combined layer by layer. The fully connected approach requires “stretching” these feature maps into a vector and then fully connecting them to each neuron in the fully connected layer. By setting the size of the convolution kernel, the input raw data are then dot-multiplied with the weight matrix of the convolution kernel and then slides to the next matrix range of the same size as the convolution kernel of the original data. This leads not only to a dramatic increase in the number of parameters, but also to a loss of spatial structure of the feature maps. In contrast, the global average pooling layer is used by averaging each feature map into an output vector. The advantage of the global averaging pooling layer is that it effectively reduces the number of parameters of the model, alleviates the computational effort, and reduces the probability of overfitting occurrence to a certain extent. After calculation, the number of parameters in the initial stage of the global pose constrained network is about 0.6 M, which is much less compared to DeepPose.

### 4.2. Results of Intelligent Analysis of Sports Health Big Data

**Figure 9:**
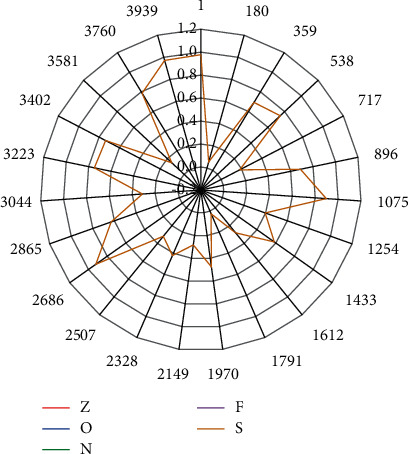
Comparison of the original EEG signals of the five types of samples.

Figure 9 shows a comparison of these five signals. The amplitude of the EEG signal during seizures is clearly in an abnormal state. From a local perspective, the sample shapes during seizures have larger amplitudes and are characterized by shape, and the energy of the seizure signals is larger overall.

It can be seen in Figure 9 that the samples of different categories differ significantly in terms of waveforms and show periodicity. These shape features can be well extracted with only a short convolution kernel, and pooling can be used to reduce the error in the location information. Next, the energy spectrum of each type of EEG shows a high energy signal during seizures. The energy spectrum of the healthy EEG shows that the EEG signal is low in energy. The energy spectrum from epileptic patients with nonseizures shows that the energy of the signal is very low. Therefore, the energy information of the signal is the feature to focus on. In previous convolutional neural networks, the convolutional kernel abstracts the overall features by deepening the number of layers. In this paper, we propose a method for energy features that can directly extend the perceptual domain of the convolutional kernel and use a huge length of the convolutional kernel to extract the energy features of epileptic EEG.

The overall architecture of the platform is divided into six layers. The activation functions not only simulate the characteristics of biological neurons, but also have excellent mathematical significance. The following is a description of what is included in each layer. It mainly collects data on students' physical health sources. Data integration layer: the data integration layer completely migrates all student physical health source data from all data resource layers to the Hadoop platform and rebuilds it, storing and managing all student physical health databases in a unified manner, providing data resources for real-time statistical analysis and multidimensional analysis of all student physical health data. In this paper, Sqoop is used to import the structured student physical health data into Hive. Data storage layer: the big data platform uses HDFS and Hbase to store student physical health data.

**Figure 10:**
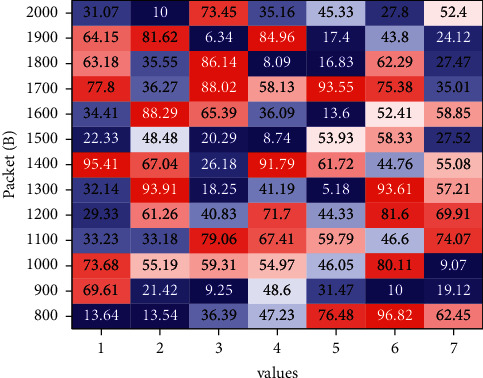
Effect of different packet sizes and transmission rates on the average delay.

The system's large amount of data analysis is mainly through a large number of students in a variety of physical examination of a large number of data results in information for systematic processing. By query in a visual form, people can directly view these visual graphics at any time, and through intuitive and accurate understanding of the student's physical and mental health, each of the following charts show the use of Hadoop technology for its design and implementation. The system functions and visualizations are shown below. The table of the number of people in an age group and the proportion of people in the total number of owners is quickly obtained by entering their age, as shown in Figure 10.

The system interface was tested and the pages were displayed well on the computer side, and the backend was logged in with three different browsers. Both traditional artificial neural networks and convolutional neural networks have introduced activation functions. The test results show that the test pages in three different kernel browsers include the main login page, the general page of physical test data management, the general page of physical test data analysis, and the permission management page. After the test, the pages can be displayed normally; the pages are not deformed; the navigation menu can be clicked normally; the page style, the overall style, is uniform, beautiful, and elegant; and the interface can meet the user experience.

After the entry of student physical test data is completed in the front-end through form input or excel import according to the system rules, the backend database can save the student physical test data correctly. When the necessary information in the data entry is incomplete or the data are not entered in the correct format as required by the template, the entry will fail and the system will pop up a warning screen to prompt the user to reenter the data in the correct format; otherwise, the database cannot save the entered data.

## 5. Conclusion

We tried to combine CNN with EEG data and found that it can be well recognized for classification and has good performance. However, compared with other structures, the performance is not very good because the accuracy is only 65.11% of the result and the accuracy is improved, but on the computational side compared with other structures, the computational complexity is reduced. It is a lightweight computation, and the lower computational complexity can guide portable devices. The objective function is the function that the model needs to optimize in the end, and usually some regular term constraints are added after the loss function. Shortly, with the rapid development of hardware configuration, we will apply real-time BCI based on motion imaging to further improve our model accuracy to validate its robustness and efficiency. Through the design and implementation of the student physical health big data platform, we can strongly promote the improvement of student physical health and reflect the trend of student physical health in time. Student physical health is the lifeblood of the future development of the country. Applying contemporary advanced information technology tools such as big data to the field of student physical health testing can better optimize the workflow of student physical health testing, as well as bring into play the potential value of student physical health data in a timely and efficient manner, providing personalized, real-time reports on student physical health to the five types of user groups of the platform.

## Data Availability

The data used to support the findings of this study are available from the corresponding author upon request.

## References

[1] Gupta S., Kaur M., Lakra S., Dixit Y. (2020). A comparative theoretical and empirical analysis of machine learning algorithms. *Webology*.

[2] Rodríguez-Ruiz J. G., Galván-Tejada C. E., Vázquez-Reyes S., Galván-Tejada J. I. H. (2020). Classification of depressive episodes using night time data; a multivariate and univariate analysis. *Programming and Computer Software*.

[3] Zheng Y., Zhao X., Yao L. (2020). Mixture kernel density estimation and remedied correlation matrix on the EEG-based copula model for the assessment of visual discomfort. *Cognitive Computation*.

[4] Akbari H., Sadiq M. T., Rehman A. U. (2021). Classification of normal and depressed EEG signals based on centered correntropy of rhythms in empirical wavelet transform domain. *Health Information Science and Systems*.

[5] Shin J., Gierl M. J. (2021). More efficient processes for creating automated essay scoring frameworks: a demonstration of two algorithms. *Language Testing*.

[6] Pimentel J. S., Ospina R., Ara A. (2021). Learning time acceleration in support vector regression: a case study in educational data mining. *Stat*.

[7] ElOuassif B., Idri A., Hosni M., Abran A. (2021). Classification techniques in breast cancer diagnosis: a systematic literature review. *Computer Methods in Biomechanics and Biomedical Engineering: Imaging & Visualization*.

[8] Peiqing H. (2022). Multidimensional state data reduction and evaluation of college students’ mental health based on SVM. *Journal of Mathematics*.

[9] Suh Y. A., Yim M.-S. (2020). A worker’s fitness-for-duty status identification based on biosignals to reduce human error in nuclear power plants. *Nuclear Technology*.

[10] Wu J., Guo P., Cheng Y., Zhu H. X.-B. X. (2020). Ensemble generalized multiclass support-vector-machine-based health evaluation of complex degradation systems. *IEEE*.

[11] Baghdadi A., Aribi Y., Fourati R., Halouani N. P. A. (2021). Psychological stimulation for anxious states detection based on EEG-related features. *Journal of Ambient Intelligence and Humanized Computing*.

[12] Zemmal N., Azizi N., Sellami M., Cheriguene S. A. M. N. (2020). Particle swarm optimization based swarm intelligence for active learning improvement: application on medical data classification. *Cognitive Computation*.

[13] Rahman J. S., Gedeon T., Caldwell S., Jones R. Z. (2021). Towards effective music therapy for mental health care using machine learning tools: human affective reasoning and music genres. *Journal of Artificial Intelligence and Soft Computing Research*.

[14] Zhao K., Ding Y., Han Y., Fan Y. (2020). Independent and reproducible hippocampal radiomic biomarkers for multisite Alzheimer’s disease: diagnosis, longitudinal progress and biological basis. *Science Bulletin*.

[15] Barenholtz E., Fitzgerald N. D., Hahn W. E. (2020). Machine-learning approaches to substance-abuse research: emerging trends and their implications. *Current Opinion in Psychiatry*.

[16] Kumari P., Gupta P., Piyoosh A. K., Tyagi B. P. (2021). Covid 19: impact on mental health of graduating and post graduating students. *Journal of Statistics & Management Systems*.

[17] Dodell-Feder D., Tully L. M., Dudek E., Hooker C. I. (2021). The representation of mental state information in schizophrenia and first-degree relatives: a multivariate pattern analysis of fMRI data. *Social Cognitive and Affective Neuroscience*.

[18] Mohan P., Paramasivam I. (2021). Feature reduction using SVM-RFE technique to detect autism spectrum disorder. *Evolutionary Intelligence*.

[19] Yang S., Xiong H., Xu K., Wang L. J. Z. (2021). Improving covariance-regularized discriminant analysis for EHR-based predictive analytics of diseases. *Applied Intelligence*.

[20] Hu W., Huang G., Li L., Zhang L. Z. Z. (2020). Video‐triggered EEG‐emotion public databases and current methods: a survey. *Brain Science Advances*.

